# Service-integration approaches for families with low income: a Families First Edmonton, community-based, randomized, controlled trial

**DOI:** 10.1186/s13063-016-1444-8

**Published:** 2016-07-22

**Authors:** Jane Drummond, Natasha Wiebe, Sylvia So, Laurie Schnirner, Jeffrey Bisanz, Deanna L. Williamson, Maria Mayan, Laura Templeton, Konrad Fassbender, for the Community-University Partnership for the Study of Children, Youth, and Families

**Affiliations:** Faculty of Nursing, University of Alberta, Edmonton Clinic Health Academy, 11405 87 Ave NW, Edmonton, T6G 1C9 Alberta Canada; Department of Medicine, University of Alberta, Edmonton, Canada; Alberta Health Services, Edmonton, Canada; Faculty of Extension, University of Alberta, Edmonton, Canada; Department of Psychology, University of Alberta, Edmonton, Canada; Department of Human Ecology, University of Alberta, Edmonton, Canada; Department of Oncology, University of Alberta, Edmonton, Canada

## Abstract

**Background:**

Increasing access to health and social services through service-integration approaches may provide a direct and sustainable way to improve health and social outcomes in low-income families.

**Methods:**

We did a community-based randomized trial evaluating the effects of two service-integration practices (healthy family lifestyle and recreational activities for children) among low-income families in Alberta, Canada. These two practices in combination formed four groups: Self-Directed (no intervention), Family Healthy Lifestyle, Family Recreation, and Comprehensive (Family Healthy Lifestyle plus Family Recreation programs). The primary outcome was the total number of service linkages.

**Results:**

We randomized 1168 families, 50 % of which were retained through the last follow-up visit. The number of service linkages for all three intervention groups was not significantly different from the number of linkages in the Self-Directed group (Comprehensive 1.15 (95 % CI 0.98–1.35), Family Healthy Lifestyle 1.17 (0.99–1.38), and Family Recreation 1.12 (0.95–1.32) rate ratios). However, when we explored the number of linkages by the categories of linkages, we found significantly more healthcare service linkages in the Comprehensive group compared to the Self-Directed group (1.27 (1.06–1.51)) and significantly more linkages with child-development services in the Family Healthy Lifestyle group compared to the Self-Directed group (3.27 (1.59-6.74)). The monthly hours of direct intervention was much lower than the assigned number of hours (ranging from 5 to 32 % of the assigned hours).

**Conclusions:**

Our findings are relevant to two challenges faced by policymakers and funders. First, if funds are to be expended on service-integration approaches, then, given the lack of intervention fidelity found in this study, policymakers need to insist, and therefore fund a) a well-described practice, b) auditing of that practice, c) retention of family participants, and d) examination of family use and outcomes. Second, if child-development services are widely required and are difficult for low-income families to access, then current policy needs to be examined.

**Trial registration:**

ClinicalTrials.gov, NCT00705328. Registered on 24 June 2008.

**Electronic supplementary material:**

The online version of this article (doi:10.1186/s13063-016-1444-8) contains supplementary material, which is available to authorized users.

## Background

An important goal for modern societies must be to develop cost-effective service-delivery policies and practices that reduce barriers between essential health and social services and families in need. Current evidence [1–3] indicates that increasing access to service through service-integration approaches may provide a direct and sustainable way to improve health and social outcomes in low-income families.

Families First Edmonton (FFE), a partnership consisting of 12 community/government organizations and an interdisciplinary research team in Edmonton, Alberta, Canada, developed this trial of service integration. Alberta has a resource-based boom-and-bust economy with Canada’s widest gap between the rich and poor [4], and during the 2007-2009 recession, the low-income population of this area grew the fastest [5], particularly in Edmonton, the provincial capital. In this region, government funding is distributed to private service-delivery agencies through a competitive process. Success of these programs is often measured in simple outputs such as the number of families enrolled. This practice contributes to a fragmented system with little collaboration and multiple barriers at every level [6]. FFE chose not to add to the fragmentation by creating a new service; instead, our aim was to optimize linkages among existing services.

We used the award-winning Canadian study by Browne and colleagues, entitled *When the Bough Breaks* (WTBB) [7] as a foundation. Browne et al. examined the outcomes of proactive comprehensive care (health promotion, employment training, and recreation activities for children) for low-income single-parent families on social assistance in the Canadian city of Hamilton, Ontario. Findings showed that families who received comprehensive service had better child, maternal, and family health outcomes than self-directed families in the control group. In addition, 15 % more families who received comprehensive service than those in the control group exited from social assistance within 1 year. These outcomes resulted in substantial savings to society in terms of social assistance payouts and reductions in (costly) emergency-service use.

FFE was formed to test whether the WTBB approach would be effective under different and more general conditions. First, assistance was provided by paraprofessionals rather than health professionals (e.g., nurses and social workers)—a change that could make this approach more affordable. Second, service delivery was implemented and data collected over a multiyear period, compared to just 1 year in WTBB, so that longer-term effects could be assessed. Third, service-delivery models that were used in FFE were similar to WTBB but were more transparent, appropriate, and sustainable in the local policy and practice environments. Fourth, interventions were administered by community-based agencies, not by a research team, a condition that was more realistic for everyday applications than was the case with WTBB.

The FFE trial was designed to a) provide clear evidence for health and social policymakers about the influence of service-delivery models and practices on enhancing and sustaining low-income families linking to existing health and social services and b) generate the types of knowledge that are essential if these service-delivery models and practices are to be adapted and implemented elsewhere. The primary research question is as follows: What are the effects of two community-based service delivery vehicles (healthy family lifestyle and recreational activities for children) compared to existing services on the number of linkages that families initiate and maintain with established health and social services?

## Methods

The full details of the protocol for this community-based randomized trial have been previously reported [8] in accordance with the CONSORT guidelines [9]. The protocol was registered with ClinicalTrials.gov (NCT00705328).

### Participant families

Families were recruited by mail, referral, and community-engagement approaches from January 2006 to January 2008 in Edmonton, Alberta, Canada. Families were eligible if a) they included at least one resident child younger than 12 years of age and b) they accessed at least one of five government assistance programs: Income Support (welfare) [10], Alberta Child Health Benefits [11], City of Edmonton’s Leisure Access [12], Alberta Adult Health Benefit [13], and Capital Region Housing [14]. Families were allowed to self-identify, resulting in diverse structures and compositions including biological two-parent families, single-parent families, adoptive families, and grandparent-led families.

The study protocol approval came from a special interdisciplinary ethics committee at the University of Alberta and the Director of the Human Research Protections Office (file number Pro00000144). Interested families contacted the community-based research office to learn about the study and ask questions. Families in which English was not a first language were offered the option of an interpreter during all interactions (e.g., on the phone while booking appointments, during the consent process, and during data collection). At the first home visit, each family was given written information about the study, which the data collector reviewed verbally. Written informed consent was obtained prior to study entry and data collection. Families were given an honorarium at each data collection period ($25 in early data-collection periods and $30 at the final data-collection period).

One adult respondent per family (“primary parent”) was selected based on familiarity with the children. To measure child outcomes, one “focus child” was randomly selected among the children within the household at screening. Limiting the measurement to one child per family was necessary to limit the measurement burden on families and reduce costs to the project. Families were ineligible if a) they could not commit to a 5-year data collection period, b) they were unwilling to allow the researchers access to the focus child, and c) a relevant interpreter was not available for families that did not speak English well. After families were recruited, they were free to withdraw from the study at any time and were no longer eligible to participate if they moved outside of Edmonton.

### Interventions

The investigators, in collaboration with community partners, developed three parameters for the service delivery models: 1) the service-integration foci of family recreation and family healthy lifestyle; 2) the practice principles of family-centeredness, cultural sensitivity, capacity building, and reflection; and 3) the hours of direct service. These parameters formed the foundation of the two service-delivery models tested: Family Healthy Lifestyle and Family Recreation. Full details of the interventions and their development are described in Table 1 using the Template for Intervention Description and Replication (TIDieR) checklist [15]. Qualitative analyses of the development and implementation of these interventions have been published [16–18].

**Table 1:** Description of FFE intervention using the TIDieR Checklist

Item	Description
Service names and elements	Community-based service-integration practice models using two vehicles (Family Healthy Lifestyle and Family Recreation); four practice principles (the practice principles of family-centeredness, cultural sensitivity, capacity building, and reflection); and a direct-service dose with low intensity (1.4 to 4.6 hours) and long duration (18 months). The vehicles and practice principles are well described in the Families First Edmonton Toolkit [35] on pages 5–6. In summary, Family Recreation focused on developing awareness, knowledge, skills, attitudes and material resources for linking to existing recreation services, and Family Healthy Lifestyle focused on developing awareness, knowledge, skills, attitudes and material resources for linking to existing social, primary health, and child/education services.
Goal and rationale	Goal was to increase use of existing service programming by low-income families with children.
	Vehicle selection was influenced by two interventions used in the comprehensive intervention of WTBB, namely health promotion and recreation. These vehicles were the health and/or recreation backgrounds against which family workers were to inform and model the problem solving, communication and resource management required to access the services needed by the families.
	Practice principles were based on a systematic review of effective interventions for school-aged children. An analysis of the 29 reviews of 1102 intervention studies [37] showed that successful programs have seven characteristics:
	1. They were holistic and integrated. The complexity of the life of the child, parent, and family is addressed.
	2. Successful programs resulted from collaborations that are multilevel and multisectoral.
	3. Successful programs were capacity building, rather than focusing exclusively on eliminating undesirable problems and behaviors.
	4. Successful programs were client-centered.
	5. Successful programs were community-based in what is available and situated in families’ neighborhoods.
	6. Successful programs were long-term, engaging long enough to show effects and enabling relationships between staff and participants to develop.
	7. Successful programs were well staffed with supportive personnel who are culturally similar to the population served.
	The amount of direct service was constrained by two items: a) the desire to evaluate the effect of a small service-integration intervention on the use of existing service and b) financial considerations, since the community partners were funding the intervention (e.g., what would be reasonably fundable for long-term implementation should positive outcomes be found). For these reasons, Family Recreation was funded for 1.4 hours per month per participant family, Family Healthy Lifestyle was funded for 3.5 hours per month, and Comprehensive (Family Recreation plus Family Healthy Lifestyle) was funded for 4.6 hours per month.
	Service-delivery vehicles, principles, and dose elements were described in a logic model and transformed into a Request for Proposals that was issued to community agencies that provided family programming. Community-based practice delivery for FFE was competitively awarded to a collaboration of four community service agencies that called their involvement in the trial “Families Matter.”
Materials and practices	Service-delivery practices and the Families Matter program support practices are fully described in the Families First Edmonton Toolkit [38].
Methods used to describe and monitor the practices	Community-based intervention, when delivered through a research project, risks losing intervention fidelity for at least two reasons: (a) use of general practice principles and very broadly identified content area within which to practice and (b) intervention drift [39]. In addition, a culturally based reluctance by service providers to submit to rigorous oversight of community-developed practices exists.
	For these reasons, action research methods were used to record and monitor the development and delivery of the service integration. An administrative database was jointly developed to include qualitative and quantitative methods of recording practice to be used to calculate dose and to audit practices.
	In addition, the administrative and supervisory staff of Families Matter met weekly with the researchers to review and apply the elements of the FFE service-delivery logic model, which built the relationship and internalized the need for intervention fidelity.
	At the same time, a researcher spent half a day each week with the supervisors and family workers, focusing on trouble-shooting the practices associated with recording the practices in the database and on the need for fidelity to the three service-integration groups.
	Families Matter also assigned family workers and supervisors to only one service-integration approach in order to support intervention fidelity.
	Lastly, focus groups and individual interviews were held, with supervisors and family workers, over the course of the 18 months of service delivery in order to specify the practices used in service integration.
Providers	Three types of providers were funded. The one manager was a professionally educated social worker. The three supervisors of the vehicles (Family Healthy Lifestyle, Family Recreation and Comprehensive) were nonprofessional and baccalaureate-educated. All of the family workers were college-educated paraprofessionals or gifted graduates of service programs delivered by the collaborating service agencies.
	The Families Matter collaborative used an in-house, ongoing schedule of training. Supervisors used reflective practice approaches with family workers. All are described on pages 20–22 of the Families First Edmonton Toolkit.
Modes and location of service delivery	Family workers used three ways to contact and work with families: face-to-face meetings in homes or other safe locations, telephone conversations, and email communication. Occasionally, family workers accompanied families to selected service visits that were counted as face-to-face meetings.
How much service and tailoring	To distribute the intensity and duration of the dose for each intervention, Families Matter used case management by the supervisors as supervised by the manager. The case management practices and protocols are described on page 17 of the Families First Edmonton Toolkit.
Modifications	As described above, action research methods were used to describe the practices developed by Families Matter to deliver service integration using four principles: family-centeredness, cultural sensitivity, capacity building, and reflection. The action research observations lead to the production of a practice model called Service-integration Flow that had eight practices and four pillars. They are described on pages 6–10 of the Families First Edmonton Toolkit.
Intervention fidelity	The interventions as delivered were different from the interventions as planned. Six families received a different intervention than the one assigned: five families received the Comprehensive intervention rather than Family Recreation (*n* = 2), Family Healthy Lifestyle (*n* = 1), and Self-Directed interventions (*n* = 1), and one family received the Family Healthy Lifestyle rather than the Self-Directed intervention. The monthly hours per family of direct FFE intervention was low (ranging from 5 to 32 % of the assigned hours) and had little variability across groups (Table 3). In addition, the Comprehensive group received about a third of the Family Healthy Lifestyle intervention contacts compared to the Family Healthy Lifestyle group (Table 3).

*TIDieR* Template for Intervention Description and Replication, *WTBB* When the Bough Breaks, *FFE* Family First Edmonton

### Trial design

Participant families were randomized to four groups: Self-Directed (no intervention), Family Healthy Lifestyle, Family Recreation, and Comprehensive (Family Healthy Lifestyle plus Family Recreation programs). Families were randomized after baseline-data collection using a 1:1:1:1 allocation ratio. Computer-generated lists of permuted blocks of eight and 12 were created by a statistician. In order to limit the number of stratification variables [19], we selected the two variables directly involved in the selection of the families. Randomization lists were stratified by type of financial support (employment income versus income support or other government assistance programs) and age of the randomly selected focus child (0 to 3.9, 4 to 6.9, 7 to 9.9, or 10 to 12.9 years). Intervention assignment was concealed in sequentially numbered, opaque, sealed envelopes. Data collectors, who went to families’ homes, were blind to intervention assignment. Research assistants, who performed and explained the random intervention assignment and scheduled interviews, intervention providers, and families were not blind to intervention assignments. Families who were assigned to the intervention groups received up to 24 months of service-integration intervention. All families were followed by researchers for 3 years (reduced from 5 years due to funding), with a minimum of eight face-to-face interviews for data collection: two at baseline (separated by a month), and two each at year 1, year 2, and year 3.

### Outcomes

The primary outcome was the number of total family linkages to health and social services as defined by an author-created tool called the “Family Services Inventory” (FSI). The FSI adopts a societal perspective and measures the public and private expenses for families. The FSI was developed to maximize precision and minimize participant burden and recall bias. An FSI toolkit comprised in-service training materials for data collectors, a user manual, a codebook, and a calendar that served as a memory aid to reduce participant recall bias. During annual interviews, data about family linkage use was collected. At the first visit of each data collection point (baseline, years 1, 2, and 3), data collectors explained how to use the FSI tool, and the family recorded their health and social service linkages for a month. At the second visit, approximately 1 month later, service linkages that occurred in the previous 28 days were collected. Each family service experience was considered an encounter. Repeated encounters within a single service were considered a single linkage (e.g., two family doctor visits within a 28-day period at the same clinic would count as two encounters but only one linkage).

For the purposes of the analysis reported here, the service linkages are subdivided into the following categories: basic needs, family challenges (e.g., addiction supports, parole, and mediation), child development, health care, childcare, and other services (i.e., nature of the service was unclear). These categories were developed through consultation with experienced social workers in Edmonton to capture the broad spectrum of services commonly accessed by vulnerable families. Each service is listed in only one category as shown in the Additional file 1. These categories were treated as secondary outcomes. Other outcomes were measured but are not reported in this paper. A full list of outcomes is provided in our published protocol [8].

### Family characteristics

Data on the following family characteristics were collected and described: the age and gender of the primary parent, age and gender of the focus child, family structure (number of adults and number of children), citizenship history (e.g., born in Canada (Aboriginal and not Aboriginal), immigrant, or refugee), geographic residence in Edmonton (northcentral, northwest, west, southwest, and southeast), the number of residence relocations, educational attainment (university, college, secondary school, and less than secondary school), current employment, and after-tax income. Other family characteristics were derived: child-to-adult ratio and household depth of poverty (DOP). The families’ household DOP was calculated as their annual household income after tax (from a maximum of two adults) divided by Statistics Canada’s 2005 low-income cut-offs [20], which are based on family and community sizes. The characteristics of the randomized families are presented in Table 2.

**Table 2:** Demographic characteristics of participant families by intervention groups

	Comprehensive	Family Healthy Lifestyle	Family Recreation	Self-Directed
N	293	293	291	291
Primary parent				
Age, y	35 (30,41)	35 (30,41)	35 (30,41)	34 (29,40)
Female	248 (84.6)	259 (88.4)	250 (85.9)	241 (82.8)
EQ-VAS	75 (60,85)	75 (59.5,85)	77 (60,90)	76 (60,90)
Focus child				
Age, y	6 (3,9)	6 (3,10)	6 (3,10)	6 (3,10)
Female	160 (54.6)	141 (48.1)	132 (45.4)	130 (44.7)
Family structure				
Lone parent	172 (58.7)	187 (63.8)	169 (58.1)	169 (58.1)
Number of adults	1 (1,2)	1 (1,2)	1 (1,2)	1 (1,2)
Number of children	2 (1,3)	2 (1,3)	2 (1,3)	2 (1,3)
Child-to-adult ratio [mean]	1 (1,2) [1.59]	1 (1,2) [1.60]	1 (1,2) [1.56]	1 (1,2) [1.55]
Citizenship history				
Born in Canada	179 (61.1)	184 (62.8)	172 (59.1)	177 (60.8)
Not Aboriginal	134 (45.7)	136 (46.4)	128 (44)	137 (47.1)
Aboriginal	45 (15.4)	48 (16.4)	44 (15.1)	40 (13.7)
Born outside Canada	114 (38.9)	109 (37.2)	119 (40.9)	114 (39.2)
Immigrant	97 (33.1)	93 (31.7)	98 (33.7)	99 (34)
Refugee	17 (5.8)	16 (5.5)	21 (7.2)	15 (5.2)
Recent^a^ immigrant or refugee	64 (22)	52 (17.9)	67 (23.3)	72 (24.8)
Edmonton residence				
Northcentral	161 (54.9)	173 (59)	156 (53.6)	177 (60.8)
Northwest	13 (4.4)	16 (5.5)	14 (4.8)	9 (3.1)
West	34 (11.6)	31 (10.6)	31 (10.7)	24 (8.2)
Southwest	34 (11.6)	31 (10.6)	34 (11.7)	30 (10.3)
Southeast	51 (17.4)	42 (14.3)	56 (19.2)	51 (17.5)
Number of residence relocations in the last year	0 (0,1)	0 (0,1)	0 (0,1)	0 (0,1)
Education				
University	61 (20.8)	57 (19.6)	71 (24.4)	73 (25.2)
College	59 (20.1)	58 (19.9)	63 (21.6)	60 (20.7)
Secondary school	117 (39.9)	117 (40.2)	102 (35.1)	101 (34.8)
Less than secondary school	56 (19.1)	59 (20.3)	55 (18.9)	56 (19.3)
Employment				
Income support	96 (32.8)	95 (32.4)	97 (33.3)	95 (32.6)
Working currently	153 (52.2)	141 (48.1)	134 (46.4)	124 (42.6)
Income – after tax				
Income, $1000 CAD	21.8 (14,30.7)	23.05 (15.8,30.9)	22.5 (15.1,32.25)	21 (15,33.1)
Household depth of poverty	73.09 (50.78,105.23)	77.85 (56.72,104.18)	74.78 (56.69,105.28)	73.4 (53.6,102.99)
Baseline linkages (28–day rate)	1.22 (0.51,2.63)	1.68 (0.78,3.00)	1.35 (0.60,2.73)	1.62 (0.70,3.00)

Counts (percentages) or medians (interquartile ranges), as appropriate. *EQ-VAS* EuroQol visual analog scale, *CAD* Canadian dollars

^a^In the last 5 years

### Statistical analyses

Analyses were performed using Stata/MP 11 (www.stata.com). To compare the intervention assigned to the intervention received, we measured the frequency of the intervention contact, length of intervention contact (in hours), and intensity of intervention contact (total hours divided by months of service divided by number served). In-person visits, phone conversations, messages left on voicemail, and emails were all considered forms of intervention contacts.

The number of service linkages was modeled using generalized linear mixed regression: a Poisson distribution with a log link and offset by the number of days of observation. The models were adjusted for the main and interaction effects of Family Healthy Lifestyle and Family Recreation interventions (thus defining four groups), baseline services linkage rate, income group (income support versus other), and the visit year (1, 2 and 3). The visit year was modelled with two indicator variables. Effects were considered significant at *P* < 0.05.

A number of sensitivity analyses were performed. First, the data were analyzed according to the intervention the participating families received (“per protocol”) rather than the intervention they were assigned (“intention-to-treat”). In addition, to account for families missing before the year 1 visit, we regressed missing outcome status against family characteristics using a generalized linear mixed regression: a binomial distribution with a logistic link. Family characteristics significantly associated with missing outcome were then adjusted for in the primary outcome. To account for missing outcome data, we imputed outcome data using the last-value-carried-forward method. Visit year was modelled as a linear variable. To determine whether the association between outcome and intervention was modified by visit year, we used an interaction term for visit year and intervention group. Lastly, the number of encounters (per month) were analyzed as the dependent variable rather than the number of linkages. Childcare encounters were not included in this sensitivity analysis because their frequent use precluded the value of this measure.

### Sample size calculation

Projecting a moderate (*f* = 0.25) and even a small (*f* = 0.10) effect size (mean divided by standard deviation), given an alpha of 0.05 and a 25 % attrition rate, the trial proposed an initial sample size of 300 families per group to detect any significant difference between the four groups (power was 0.99 and 0.72, respectively). The trial randomized approximately 290 families to each group. The overall alpha value was not controlled for multiple comparisons. No interim analyses were planned.

## Results

### Trial participants

**Fig. 1:**
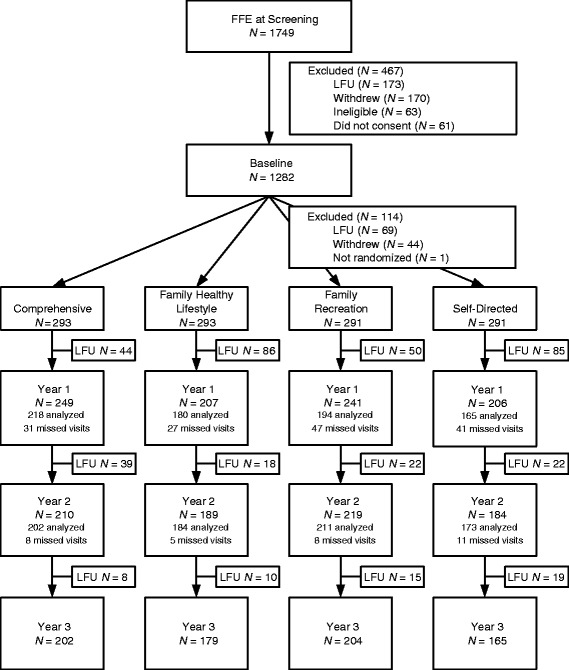
Participant family flow diagram. *LFU* loss to follow-up

Of the 1749 families screened for eligibility, 1282 families were enrolled, and 1168 were randomized: 293 to Comprehensive, 293 to Family Healthy Lifestyle, 291 to Family Recreation, and 291 to Self-Directed (Control). The remaining 114 participants were unavailable for randomization (Fig. 1). Table 2 provides descriptions of the family characteristics by assigned intervention groups. More than 85 % of the primary parents were women; the median age was 35 years. Their median health-related quality of life score as measured by EQ-VAS was 75 % [21]. The EQ-VAS is a visual analog scale used by individuals to self-report their current global health status; e.g., 100 % would indicate perfect health. Single parents headed almost 60 % of the families. Approximately 60 % of the primary parents were born in Canada. Of those, 25 % were Aboriginal. Of those not born in Canada, 15 % were refugees, and 57 % immigrated to Canada in the last 5 years. A third received income support. Almost half were currently employed. The median after-tax income was $22,000; the median household depth of poverty was 75 %.

Of those families randomized, 50 % participated in all the data-collection points (see Fig. 1). With regression analyses, we found that the following family characteristics (from Table 2) were associated with more missed data collection visits: younger primary parents, male primary parents, greater numbers of children, Northcentral residence, having relocated in the last year, lower educational attainment, and a less healthy primary parent (as measured by the EQ-VAS).

### Intervention fidelity

The interventions as delivered were different from the interventions as planned. The full details of intervention fidelity are described in Table 1 using the TIDieR checklist. In summary, the dose in each intervention was diluted and the Comprehensive intervention no longer equated to the additive components of the Family Healthy Lifestyle and Family Recreation interventions (Table 3). As such, the data are analyzed as a four-group randomized trial rather than a factorial randomized trial.

**Table 3:** Intervention fidelity by assigned interventions

	Comprehensive	Family Healthy Lifestyle	Family Recreation	Self-Directed
Assigned monthly hours of intervention				
Family Healthy Lifestyle	4.60	3.50	0	0
Family Recreation	1.40	0	1.40	0
Actual monthly hours of intervention				
Family Healthy Lifestyle	0.22 (0.15,0.30)	0.68 (0.49,1.04)	0 (0,0)	-
Family Recreation	0.29 (0.21,0.37)	0 (0,0.02)	0.45 (0.36,0.54)	-
Number of contacts				
Family Healthy Lifestyle	1.17 (0.82,1.61)	2.33 (1.85,3.50)	0 (0,0)	-
Family Recreation	2.29 (1.74,2.91)	0 (0,0.14)	1.89 (1.55,2.26)	-

Median (IQR). *IQR* interquartile range

These data were only available in 868 of 1168 families (74 %)

### Linkages with health and social services

The mean number of linkages during the 28-day baseline period were 0.05 for basic needs, 0.01 for family challenges, 0.02 for child development, 0.79 for health care, 0.19 for childcare, and 0.06 for other services.

**Fig. 2:**
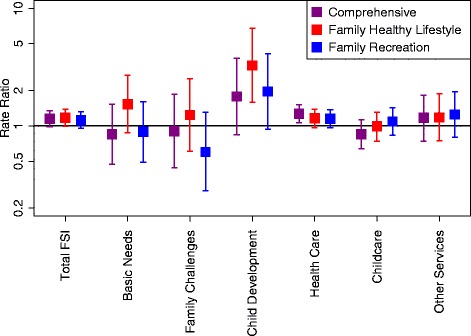
Rate ratios of family services linkages by intervention group and type of service. The *boxes* represent the point estimates of the rate ratios in family service linkages between participant families in intervention groups versus participant families in the Self-Directed group. The *whiskers* represent the upper and lower confidence limits of the rate ratios. Confidence intervals that cross the line where the horizontal rate ratio equals 1 are significant at *P* < 0.05. *Purple markers* depict the Comprehensive group (versus the Self-Directed group); *red markers* depict the Family Healthy Lifestyle group (versus the Self-Directed group); and *blue markers* represent the Family Recreation group (versus the Self-Directed group). Rate ratios are adjusted for baseline service linkage rate, income group (income support versus other), and follow-up visit year. *FSI* Family Services Inventory

Table 4 (and Fig. 2) show the rate ratios of linkages to health and social services for the Comprehensive, Family Healthy Lifestyle, and Family Recreation groups versus the Self-Directed group. All three interventions were not significantly different in terms of the total number of linkages from the Self-Directed group (Comprehensive 1.15 (95 % CI 0.98–1.35), Family Healthy Lifestyle 1.17 (0.99–1.38), and Family Recreation 1.12 (0.95–1.32) rate ratios), although all three showed a trend toward favoring an intervention.

**Table 4:** Rate ratios of family service linkages by intervention group and type of service

Outcome	Comprehensive	Family Healthy Lifestyle	Family Recreation	Self-Directed
All family services	1.15 (0.98,1.35)	1.17 (0.99,1.38)	1.12 (0.95,1.32)	1.00
Basic needs	0.85 (0.47,1.53)	1.53 (0.87,2.69)	0.89 (0.49,1.61)	1.00
Family challenges	0.90 (0.44,1.85)	1.24 (0.61,2.52)	0.60 (0.28,1.30)	1.00
Child development	1.78 (0.84,3.75)	**3.27 (1.59,6.74)**	1.96 (0.94,4.11)	1.00
Health care	**1.27 (1.06,1.51)**	1.16 (0.96,1.39)	1.15 (0.96,1.37)	1.00
Child care	0.85 (0.64,1.13)	0.99 (0.74,1.31)	1.09 (0.83,1.43)	1.00
Other services	1.17 (0.74,1.83)	1.18 (0.75,1.87)	1.25 (0.80,1.96)	1.00

Rate ratios were adjusted for the main and interaction effects of Family Healthy Lifestyle and Family Recreation interventions, baseline service linkage rate, income group (income support versus other), and offset by the days of follow-up within each visit (≤28 days). Significant differences at *P* < 0.05 between intervention groups and the Self-Directed group are in *bold*

For the specific types of services, the rate ratios were not significantly different between interventions groups and the Self-Directed group except for two. Significantly more healthcare service linkages occurred in the Comprehensive group compared to the Self-Directed group (1.27 (1.06–1.51)), and significantly more linkages occurred with child-development services in the Family Healthy Lifestyle group compared to the Self-Directed group (3.27 (1.59–6.74)).

### Sensitivity analyses

When participant families were analyzed according to the intervention they received rather than the intervention they were assigned, the rate ratio of total service linkages did not change (Additional file 2: Table S1). The results were similar when we imputed missing outcome data with previous visit outcome data and when we adjusted the results for the covariates (younger and male primary parents, greater number of children, Northcentral Edmonton residence, relocations in the last year, less education, and lower health score for the primary parent according to the EQ-VAS) associated with the missing outcome data.

When the number of encounters rather than the number of linkages were modelled, the rate ratio was attenuated for the Comprehensive group compared to the Self-Directed group (Additional file 2: Table S1). The results for Family Healthy Lifestyle were slightly attenuated, and the results for Family Recreation remained similar.

In Additional file 2: Figure S1, the top panel shows the observed mean rates for each randomized group over the time points (baseline, and years 1, 2, and 3). In general, all groups decreased their rate of service linkage between baseline and year 1 (*P* < 0.001), and then, the rates (including the rate from the Self-Directed group) were attenuated between years 1 and 2 and between years 2 and 3. Since the baseline rates for Family Healthy Lifestyle and Self-Directed groups are greater than those for the Family Recreation and Comprehensive groups, we show the mean rates over time adjusted for the baseline rate (Additional file 2: Figure S1).

When the year of the visit was modelled as a linear variable rather than two indicator variables, the results did not change (Additional file 2: Table S1). In order to assess the impact of the year of the visit, following the initiation of the intervention (which occurred over a median of 22 months), we tested the interaction between the year of the visit and the interventions. None of the interactions were significant (data not shown).

## Discussion

In summary, neither Family Healthy Lifestyle nor Family Recreation significantly increased the total number of linkages to established health and social services. While the number of linkages increased for all three community-based service-integration groups compared to the Self-Directed group (Additional file 2: Figure S1), the absolute increase in the number of linkages was small (15 % for Comprehensive, 17 % Family Healthy Lifestyle, and 12 % for Family Recreation). The unadjusted 28-day rate at year 3 for the Self-Directed group was 0.09 or about 12 linkages over a year. A 15 % increase over that would be 14 linkages per year or two additional linkages per year per family. Depending on the downstream benefits and costs, this modest increase in service access may be important.

Contrary to the findings of WTBB [7], our Comprehensive group did not show better results than the other three groups. This may be due to a number of reasons. First, unlike WTBB, we did not include employment retraining in the Family Healthy Lifestyle intervention. Second, our Comprehensive group received less than half of the planned Family Healthy Lifestyle intervention. Third, our primary outcome was the number of all service linkages rather than the number of participants on social assistance or costs. Finally, our sample differed considerably from the WTBB sample. For example, the WTBB families were all single-parented (versus 60 % in FFE), all were on social assistance (versus 33 % in FFE), and most were Canadian born and non-Aboriginal (versus inclusion of Aboriginal (16 %), immigrant (33 %) and refugee participants (6 %)).

When we consider the categories of services in our study, we see a significant and large threefold increase in linkage to child-development services in the Family Healthy Lifestyle group and a modest, but significant, 27 % increase in accessing health services in the Comprehensive group. While the latter may be a false-positive result due to the number of secondary outcomes and the number of intervention groups, given the magnitude of the former result, the result is likely a true positive. This finding supports the targeting of child-development services through service-integration approaches but should be considered against the need for developmental programming in Alberta. We know that in Alberta [22], more than one in five children has been diagnosed with a disability by the time the children reach kindergarten, and a further one in five children, with no reported disabilities in kindergarten, have difficulty in one or more areas of development. Evidence shows that children living in low-income situations often have developmental challenges that are hard to counter without services. The presence of child-development challenges increase the complexity in families and makes meeting other needs more difficult [23–25].

The supervisor of the Healthy Family Lifestyle approach was highly skilled in the use of reflective practice and addressed child-development issues before other family challenges. We determined that the number of contacts required to increase a family’s child-development linkages by one was 23 per month. This intensity is likely required for a number of reasons. First, numerous contacts are required to break down the stigma parents experience when child-development issues are identified [26]. In addition, because child-development services are typically part of the private system, it takes time to find ways to subsidize the payment for the services. Finally, several contacts are likely required to simply search out the available child-development services [27]. Given the Alberta provincial context, where 40 % of kindergarten students have been diagnosed and/or screened to be at-risk for developmental delays, using the FFE strategy of finding and convincing low-income families to use expensive child-development services may not be the best approach. More likely, the challenge for Alberta policymakers is to either develop effective population screening to accompany accessible (acceptable, inexpensive, and plentiful) child-development services or develop and provide universal early child-development programming and make it easily accessible to low-income populations.

Finally, it is useful to consider the lack of effect of these service-integration vehicles on linkages to already established basic needs, family challenges, and childcare services between the categories of intervention in this study. In our experience, food insecurity, poor housing, and available childcare are big challenges for participants in all groups. Therefore, in the case of basic needs and childcare, a ceiling effect due to pre-existing full engagement with the available, often both limited and partial [28, 29], services is likely operating. This would mean that service-integration approaches might not expand linkages in these areas. Policymakers should continue increasing the availability of adequate housing, fresh/healthy food, and childcare for low-income families. It is unlikely that the lack of effect on support services for family challenges is explained by the full engagement in services available. More probably, family priority, stigma, expense, and lack of access contributed to the underutilization of family support services across the study groups [17, 30, 31].

### Limitations

Along with a threefold increase in linkage to child-development services, the strengths of our study include the diversity of our families across ethnicity, citizenship, family structure, education, employment and income, and a measured quality of life for low-income parents that can be compared to that of any number of populations. An EQ-VAS score of 75 shows that low-income parents on average display a highly compromised health state [32].

Our study also had a number of limitations. First, as previously mentioned, neither the full dose nor the ratio of doses between intervention groups was delivered as planned. Thus, the size of effect was expected to be lower and since the groups could no longer be collapsed in order to carry out the planned 2 x 2 factorial trial, there was less statistical power. The origins of this dose dilution are found in three areas related to the decision to deliver the service-integration approaches through a collaboration of existing agencies. One, social service agencies are used to being evaluated on the numbers of families served, not on the quantity or quality of their practice nor on the outcomes from that practice. Two, and related to the first, practice is often principle-based, as these service-integration vehicles were as opposed to the describable best behavioral practices upon which the practitioner, supervisor, and family can reflect. Three, professionals (nurses and likely social workers) are more likely than paraprofessionals to persist in delivering behavioral practices with low-income families [33, 34]. Constraints on public funding have led to the use of paraprofessional staff in the delivery of community-based service. These workers are poorly paid and thus turnover in employment is high, with expensive retraining required for new staff. They also need more skilled supervision because they are less persistent than professional workers.

A second limitation is that the interventionists were matched to the three intervention groups rather than to the two service-integration vehicles, each with a separate supervisor. The rationale was that the former approach allows the workers to focus on building relationships with their families so that more accurate assessments could be made and more teachable moments optimized. Such an approach should have given the Comprehensive group an advantage because they could build relationships using the relatively nonthreatening Family Recreation vehicle and go on to transfer and build on behaviors learned to the Family Healthy Lifestyle vehicle. Alternatively, the use of two supervisors, one for each vehicle (Family Recreation or Family Healthy Lifestyle), would have necessitated the use of two family workers for the Comprehensive group. This is not the practice of “real world” service agencies. The issue of workload and case management are practical considerations in dividing supervision. Therefore, because of the importance placed by the delivery agencies on developing enduring interpersonal relationships with clients, we may have confounded the effects of the interventions with the effects of the three intervention groups.

Third, as shown in Additional file 2: Fig. S1, our study may have exhibited a form of observer bias, a so-called Hawthorne effect [35]. The number of baseline links to services was high and then dropped for all four groups, only to climb back by the third year of measurement. The act of being observed (our data collectors while visiting and collecting data) might have temporarily decreased links to services in the first year only to return to the baseline rates over subsequent years. However, given that all four groups, including the Self-Directed group, exhibited the same downward-then-upward trend, observer bias did not likely impact the comparative effects between groups. Alternatively, in order for the families to balance their schedules, the data collection periods (the 28-day period prior to their visits) may not have overlapped sufficiently with the time allocated for intervention when the families may have accessed more services.

Fourth, since the families were not blinded, contamination bias may have diluted the effects of the interventions. Families may have transferred some of their learning to each other.

Fifth, the Family Services Inventory tool, given the short 1-month window of data captures and that the tool itself has not yet been validated, may not have adequately captured the linkages to service, thus diluting the effects of the interventions. For example, transportation supports are often received in conjunction with other services and would have been categorized with the primary service rather than as a basic need transportation service. In addition, since the data captures were retrospective, the results are subject to recall bias.

Similar to WTBB, the last limitation of our study was that 50 % of the enrolled families were lost to service and/or follow-up. Both high- and lower-functioning low-income families tend to avoid participation in social interventions such as service integration [36]. As well, low-income families tend to move frequently and are busy keeping their lives organized. The lives of lower-functioning low-income families can be complicated by issues such as mental health challenges, substance-dependency issues, and intergenerational family functioning problems. In addition, both higher- and lower-functioning low-income families are the most likely to be dropped from service by agencies or “have their files closed.” Although files were not to be closed by Families Matter in this study, losing track of “easy” families or “difficult” cases was observed to be a passive way to manage caseloads.

## Conclusions

FFE is an early and credible example of research in community practice that tried to operationalize shifts in service development into testable hypotheses and variables that could inspire practice-based researchers and policy-makers. The challenges embedded in an entrenched and siloed service-delivery sector that is under pressure to integrate and respond effectively to the effects of poverty were evident in the processes and outcomes.

### Recommendations for future research

This study was a community-based trial on the effectiveness of principle-based practices for linkage to services by low-income families. The poor intervention fidelity in this study emphasized the existing challenges in modern community-based service delivery. Research is required to determine the processes the service-delivery sector needs to have in place in order to support full delivery of service practices. Consideration should be given to developing and documenting processes that provide behavioral description of practice; develop behavioral and attitudinal qualities of supervisors of paraprofessionals; improve retention of family participants; and provide early and ongoing audits of progress in the delivery of service.

### Recommendations for policy and practice

Policymakers and funders have a couple of challenges. First, if funds are to be expended on service-integration approaches, then, given the intervention fidelity found in this study, policymakers need to insist, and therefore fund a) better-described practice, b) the auditing of that practice, c) retention of family participants, and d) the examination of family service use and outcomes. Second, if child-development services are widely required and are difficult for low-income families to access, then current policy needs to be examined. The following two questions need evaluation: 1) Are scarce and stigma-laden services best accessed through the provision of service integration? and 2) Is a system of accessible universal child-development programming more useful to the long-term health and economic productivity of the population?

## Abbreviations

DOP, depth of poverty; EQ-VAS, European Quality of Life–Visual Analog Scale; FFE, Families First Edmonton; FSI, Family Services Inventory; WTBB, When the Bough Breaks

## Additional files

Additional file 1: Categorization and examples of Edmonton services. (PDF 235 kb) Additional file 2: Table S1. Relative rates of all family services linkages by intervention group: Sensitivity analysis, and **Figure S1.** Rates of family services linkages by intervention group over time. (PDF 20 kb)
