# Supplementary material for: Service-integration approaches for families with low income: a Families First Edmonton, community-based, randomized, controlled trial
Source: Trials. 2016 Jul 22;17:343. doi: 10.1186/s13063-016-1444-8 (PMC4957834; doi:10.1186/s13063-016-1444-8)
Supplement: Additional file 1: — Categorization and examples of Edmonton services. (PDF 235 kb) [file 13063_2016_1444_MOESM1_ESM.pdf]

Basic Needs

Social services that help families address material shortages such as:

Food
Clothing
Housing/Shelter (excluding emergency shelters for domestic violence situations)

This typology also includes government subsidy programs for low-income families with children as these subsidies help families cover the cost of basic needs.

Examples of "Basic Needs" service providers:

Alberta Adult Health Benefit
Alberta Child Health Benefit
Alberta Works - Child Support Services
Assured Income for the Severely Disabled
Basic Shelf Program
Bissell Centre
Boyle Street Co-Op/Boyle Street Community Services
Bread Run
Canada Revenue Agency (Alberta Family Employment Tax Credit)
Canadian Native Friendship Centre
Capital Region Housing Corporation
Child Care Subsidy Program
Christmas Bureau of Edmonton
Christmas Dinner
Christmas Hampers
Clothing
Coats for Kids and Families
Community Lunches
Dickinsfield Amity House (Swap Shop)
Edmonton Catholic Schools (Provision of Basic Services)
Edmonton Gleaners Association (Food Banks)
Edmonton Inner City Housing Society
Edmonton’s School Lunch Program
Excess Food Program
Food Banks
Food Basket Depots
Food Co-op
Food Donations
Food Hampers
Food Programs

Free Clothing
Goodwill Industries of Alberta
Goodwill Thrift Shops
Habitat for Humanity Edmonton Society
Hope Terrace
Housing Supports
Marian Centre (Soup Kitchen/Bagged Lunches)
Metis Urban Housing Corporation of Alberta
Mustard Seed Street Church
Out of School Care Subsidies
Salvation Army Community and Family Services
Santa's Anonymous
Social Assistance
Social Services
Society of Learning and Community Outreach (Bread Run)
Soup Kitchens
St. Vincent de Paul Society
Student Financial Aid
Student Food Bank
Suit Yourself, the Edmonton Wardrobe for Women in Need
Supports for Independence (SFI)
Swap Shop
The Direct Rent Supplement Program
The Family Network Shelter (Basic and Emergency Services)
Thrift Stores
Toys for Tots
United Way of the Alberta Capital Region (Coats for Kids and Families)
Unity Centre of Northeast Edmonton (Food Program)
WeCan Cooperative for Community and Economic Development (Food Basket Depots)
Welfare

Family Challenges

Social services used by families who are experiencing unique challenges. These services assist families with family challenges such as:

Family violence
Addictions
Parole
Mediation services
Child services (related to the care and safety of children)

Examples of "Family Challenges" services providers:

24 hour Safety Unit (Child and Family Services Authority #6)
AADAC (Alberta Alcohol and Drug Abuse Commission)
Aboriginal Child and Family Support Program (Métis Child and Family Services Society of Edmonton)
Aboriginal Consulting Services Association (Circle of Safety)
Aboriginal Consulting Services Association (Family Violence Teaching Circle for Women)
Aboriginal Family Wellness Program (Native Counseling Services of Alberta)
Abused Women's Support Group (Mill Woods Family Resource Centre)
Addiction and Recovery Program (LDS)
Addiction Treatment Centres
Adoption Services (Alberta Government Services)
Adult Groups (Mill Woods Family Resource Centre)
Alberta Justice & Attorney General
Alberta Solicitor General and Public Security
Catholic Social Services (Family Living Program, Family-Teen Mediation)
Child Abuse Hotline
Child Protective Services (Alberta Government Services)
Child Welfare (Alberta Government Services)
Children's Services (Child Abuse and Neglect) (Alberta Government Services)
Co-Dependents Anonymous
Domestic Violence Support Group
Edmonton Chapter Alcoholics Anonymous
Edmonton Community Legal Centre
Edmonton Police Services
Family Enhancement Program (Alberta Government Services)
Family Enhancement Program (Child and Family Services Authority #6)
Family Ties program (Edmonton YMCA)
Family Violence Prevention Centre (Edmonton John Howard Society)
Lawyers
Legal Aid Society of Alberta

Making Connections Groups (Abused Women) (City of Edmonton)
Mediation Services
National Parole Board
Saint Therese Project (Grey Nuns Hospital)
Sexual Assault Centre of Edmonton
Shelters
St. Albert Stop Abuse in Families (SAIF) Society
Suicide Prevention and Crisis Line (Salvation Army)
The Mediation and Restorative Justice Centre
The Support Network
Transitions Rehabilitation Association of St. Albert and District
Understanding Anger for Women (KARA)
WINGS of Providence Society (Crisis Shelter)

Child Development

Social services that support the development of children and families. Services accessed by families include:

- Immunization and "Health for Two" programs
- Educational programs for children
- Parenting support programs
- Social and recreation focused programs for children and youth (e.g., youth drop in centres)

This typology excludes programs that have a child care component (e.g., ABC Head Start).

Examples of "Child Development" service providers:

- Abbottsfield Youth Project Society
- ASSIST Community Services Centre
- Association for Safe Alternatives in Childbirth
- Ben Calf Robe Society
- Bent Arrow Traditional Healing Society
- Big Sister and Big Brother Society of Edmonton and Area
- Boys' and Girls' Clubs of Edmonton
- Alberta Health Services and Caritas Health
  (immunization, perinatal, and Health for Two programs)
- Catholic Social Services
- Centre for Family Literacy
- Edmonton Catholic Schools (parenting classes)
- Edmonton Federation of Community Leagues
- Edmonton Public Libraries
- KARA Family Support Centre
- La Leche League
- Multicultural Health Brokers
- Native Counseling Services of Alberta
- Norwood Child and Family Resource Centre (Healthy Families program)
- Terra Centre for Pregnant and Parenting Teens (parenting program)
- Various churches (parenting classes/groups)
- Mill Woods Family Resource Centre

Health Care

Health services or services that help families access mental or physical health services.

Examples of "Health" service providers:

Acne Clinics of Canada
Alberta Blue Cross
Alberta Cancer Board
Alberta Health Care Insurance Plan
Alberta Health Services and Caritas Health
Alberta Mental Health Board
Alternative Medical Services
Alberta Health Services
Capital Health
Children's Mental Health Regional Intake
Child Psychiatry Associates
Dentists (*various*)
Edmonton Emergency Response Department
Edmonton General Continuing Care Centre
Emergency Services
Health Care Research Studies
Health Centres (*various*)
Health Food Stores (*various*)
Hys Centre
Laboratories (including Diagnostic Testing Centres)(*various*)
Medical Clinics (*various*)
Medical Specialists (*various*)
Natural Health Centres
Opticians
Oxygen Suppliers
Pharmacies (*various*)
Physiotherapists (*various*)
Poison Prevention and Control Program
Prosthetic and Orthotic Services (*various*)
Psychologists
Rehabilitation Services
St. Michael's Health Group
University of Alberta (Department of Dentistry)
Weight Loss programs (*various)*

Last Updated: January 11, 2013

Child Care

Services that provide child care. Types of services include:

All day, before, or after school care
Respite care
Child development programs if there is a child care component (e.g., ABC Head Start).

This category excludes “courtesy” child care provided by dentists or doctors.

Examples of “Child Care” services providers:

Alberta AdaptAbilities Association (Drop in Respite)
Bissell Centre (Drop-In Day Care services)
CANDORA
Catholic Social Services
Churches
Day Cares (*various*)
Day Homes (*various*)
Dickinsfield Amity House
Edmonton Catholic Schools
Edmonton Gleaners Association (Crystal Kids Youth Centre)
Edmonton Mennonite Centre for Newcomers
Edmonton YMCA (Respite, Daycares, Out of School care)
Family Linkages Foundation of Alberta (Respite)
Family support for children with disabilities (childcare support) - Alberta Government
Fulton Child Care Centre
Glengarry Child Care Society (Boys and Girls Clubs of Edmonton)
Hardisty Family Relief Services (Respite)
Jasper Place Child and Family Resource Centre (Child Care program)
KARA Family Support Centre
Kids Kottage Foundation (Crisis Nursery)
Mill Woods Recreation Centre
Multicultural Health Brokers
Oliver Centre Early Learning Programs for Children and Families Society
Persons with Developmental Disabilities (Edmonton Community Board) (Respite)
Public school daycares
Woodcroft Relief Home (Respite)
YWCA - Respite and Host Family program

Other services

Social services that did not thematically fit with the preceding categories or the service provided was unclear. Future category development may be limited due to low sample sizes.
