# Supplementary material for: Service-integration approaches for families with low income: a Families First Edmonton, community-based, randomized, controlled trial
Source: Trials. 2016 Jul 22;17:343. doi: 10.1186/s13063-016-1444-8 (PMC4957834; doi:10.1186/s13063-016-1444-8)
Supplement: Additional file 2: Table S1. — Relative rates of all family services linkages by intervention group: Sensitivity analysis, and Figure S1. Rates of family services linkages by intervention group over time. (PDF 20 kb) [file 13063_2016_1444_MOESM2_ESM.pdf]

Table S1. Relative rates of all family services linkages by intervention group: Sensitivity analyses

Sensitivity Analyses	Comprehensive	Family Healthy Lifestyle	Family Recreation	Self-Directed
Primary	1.15 (0.98,1.35)	1.17 (0.99,1.38)	1.12 (0.95,1.32)	1.00
Per protocol	1.15 (0.98,1.35)	1.17 (0.99,1.38)	1.12 (0.95,1.32)	1.00
Visit year as linear variable	1.15 (0.98,1.35)	1.17 (0.98,1.38)	1.12 (0.95,1.32)	1.00
Imputed outcome using LVCF	1.14 (0.97,1.34)	1.17 (0.99,1.38)	1.12 (0.95,1.32)	1.00
Adjusting for variables associated with missing outcome	1.15 (0.98,1.36)	1.15 (0.97,1.36)	1.14 (0.97,1.34)	1.00
Using family services encounters	1.01 (0.81,1.27)	1.11 (0.87,1.40)	1.13 (0.90,1.42)	1.00

LVCF last-value carried forward

Figure S1. Rates of family services linkages by intervention group over time

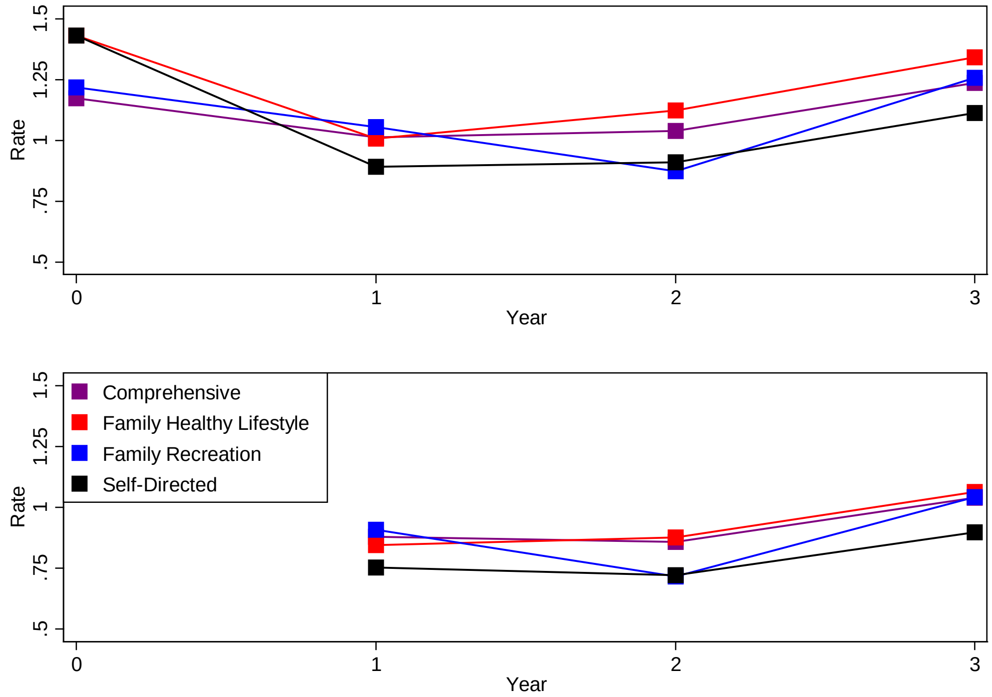

Rates of service linkages per 28 days are plotted for each timepoint (baseline [year 0], year 1, 2, and 3) by intervention group. In the top panel the rates are unadjusted. In the bottom panel the rates are adjusted for baseline rate. The rates in both panels are not adjusted for income group.

Purple markers represent the Comprehensive group; red markers represent the Family Healthy Lifestyle group; blue markers represent the Family Recreation group; and black markers represent the Self-Directed group.
